# A quantitative model of temperature-dependent diapause progression

**DOI:** 10.1073/pnas.2407057121

**Published:** 2024-08-28

**Authors:** Loke von Schmalensee, Philip Süess, Kevin T. Roberts, Karl Gotthard, Philipp Lehmann

**Affiliations:** ^a^Department of Zoology, Stockholm University, Stockholm 10691, Sweden; ^b^RT4, Climate, Ecosystems and Biodiversity, Bolin Centre for Climate Research, Stockholm University, Stockholm 10691, Sweden; ^c^Department of Animal Physiology, Zoological Institute and Museum, University of Greifswald, Greifswald 17489, Germany

**Keywords:** diapause, insect, diapause termination, thermal performance curve, ecological predictions

## Abstract

Winter diapause in insects is commonly terminated through cold exposure, which, like vernalization in plants, prevents development before spring arrives. Currently, quantitative understanding of the temperature dependence of diapause termination is limited, likely because diapause phenotypes are generally cryptic to human eyes. We introduce a methodology to tackle this challenge. By consecutively moving butterfly pupae of the species *Pieris napi* from several different cold conditions to 20 °C, we show that diapause termination proceeds as a temperature-dependent rate process, with maximal rates at relatively cold temperatures and low rates at warm and extremely cold temperatures. Further, we show that the resulting thermal reaction norm can predict *P. napi* diapause termination timing under variable temperatures. Last, we show that once diapause is terminated in *P. napi*, subsequent development follows a typical thermal performance curve, with a maximal development rate at around 31 °C and a minimum at around 2 °C. The sequence of these thermally distinct processes (diapause termination and postdiapause development) facilitates synchronous spring eclosion in nature; cold microclimates where diapause progresses quickly do not promote fast postdiapause development, allowing individuals in warmer winter microclimates to catch up, and vice versa. The unveiling of diapause termination as one temperature-dependent rate process among others promotes a parsimonious, quantitative, and predictive model, wherein winter diapause functions both as an adaptation against premature development during fall and winter and for synchrony in spring.

## Significance

Climate change has disproportionately strong effects on winter conditions, yet winter biology remains generally understudied. This is true for insect diapause, a vital winter adaptation in seasonal environments, whose continuous temperature dependence has been largely overlooked. Diapause often involves a developmental arrest that is terminated after extended cold exposure. This type of diapause presents as a binary response (in diapause or not), complicating studies of the underlying processes that resume development. We introduce a quantitative model of the temperature dependence of diapause termination and demonstrate its predictive capacity in novel thermal environments using a butterfly model system. Our approach promotes predictive models of the impact of winter climate on diapause and consequently our understanding of current and future insect responses.

Diapause is a physiological resting state that many insects enter before predictably unfavorable environmental conditions set in, like cold winters or dry summer periods (1, 2). As such, diapause constitutes a key adaptation to life in seasonal environments, dictating geographical ranges and temporal distributions of numerous insect species (3). In temperate regions, diapause is crucial for preventing the normal life cycle of insects from proceeding into the dead end of an inhospitable winter by putting development on pause (2, 4). Yet, reaction norms for the termination of this vital developmental arrest remain understudied, holding back mechanistic models of insect winter biology and thus projections of, for instance, range shifts from changes in winter climate. Fortunately, we argue, this knowledge gap is largely due to surmountable methodological challenges. We here propose a solution to these challenges, providing a quantitative and parsimonious framework for the temperature dependence of insect winter diapause.

Winter diapause is generally initiated during late summer or fall, leading to suppressed metabolism and development throughout fall and the ensuing winter (5, 6). In insects, resumption of temperature-sensitive development (i.e., termination of diapause) in time for the subsequent growth season commonly requires prolonged cold exposure, much like vernalization in plants (7–9). Indeed, qualitative models of insect “cold sum counting” have been put forth (10–12), yet no models have been developed that can generate quantitative predictions of diapause termination timing across multiple temperatures—arguably due to difficulties in measuring the exact time point at which diapause termination occurs. Because of the typically cryptic nature of diapause phenotypes, and because postdiapause development normally does not proceed under winter conditions, studying winter diapause termination usually requires both artificial winter and postwinter treatments. Individuals that develop (shortly enough) after being moved from winter to postwinter conditions are deemed to have terminated diapause. However, because diapause could have been terminated at any point in time before the transfer, quantifying termination rates and establishing their relationship to temperature is a significant challenge.

This challenge does not apply to many other important biological rate processes, like development, for which metamorphosis timing can be directly observed, or growth, for which mass can be recorded continuously (13). For such traits, individual rates can be measured at different temperatures, and thermal reaction norms can be fitted directly to the data. The resulting thermal reaction norms can subsequently be used to predict rates under novel, and even variable, temperature conditions (14). There is no a priori reason that the often-clear influence of temperature on the rate of diapause termination should not, too, be continuous and definable by a thermal reaction norm. Indeed, the hypothetical idea of diapause progression as a continuous, unimodal, temperature-dependent response was first discussed by Andrewartha already in 1952 (15), but empirical advances on the topic have since been lacking, with two notable exceptions. In 1997, a study by Johnsen et al. (16) showed that an indirectly parameterized three-stage model of diapause initiation, diapause termination, and postdiapause development corresponded relatively well with empirical emergence dates. More recently, Toxopeus et al. (10) found phenomenological support for unimodal thermal reaction norms for diapause progression in the fly *Rhagoletis pomonella*. Yet, explicitly estimated thermal reaction norms for diapause progression require multiple temperature treatments and are, as Toxopeus et al. underline, still lacking. Likely, the reason for why no such responses have yet been quantified lies in diapause’s apparently binary nature and the cryptic transition to a postdiapause developmental stage with a different thermal sensitivity (15).

**Fig. 1.**
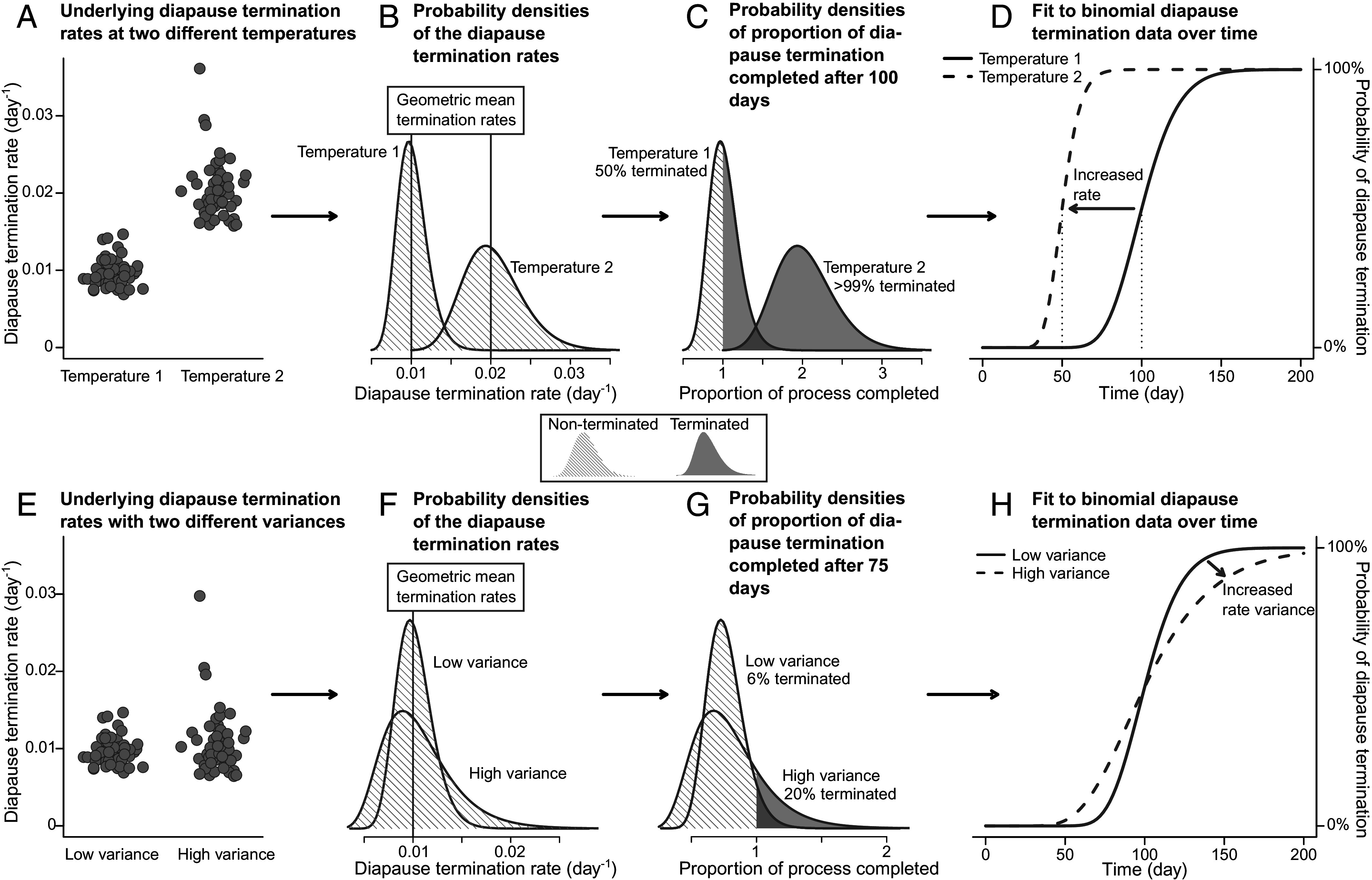
Conceptual figure showing how a continuous, log-normal, diapause termination rate will result in binomial diapause termination data, and, consequently, how the distribution of rates can be inferred from the data for a given temperature. The *Upper* panels (*A–D*) show two of simulated lognormal distributions of diapause termination rates with different geometric means, but the same variance. The *Lower* panels (*E–H*) show two simulated lognormal distributions of diapause termination rates with different variances, but the same geometric mean. *Left* panels (*A* and *E*) show the simulated diapause termination rates. Panels in the second column (*B* and *F*) show the probability densities of these distributions. Panels in the third column (*C* and *G*) show the probability densities of summed rates over a number of days (100 d in panel *C*, 75 d in panel *G*), where gray areas represent individuals that have completed the termination process (summed termination rate > 1). *Right* panels (*D* and *H*) show how changes in the underlying distribution of termination rates will influence the cumulative distribution functions that describe the binomial diapause termination data of over time.

This problem can be circumvented by studying diapause termination at the level of (statistical) populations. By consecutively transferring individuals from winter to postwinter conditions, the relationship between diapause termination and time under winter conditions can be established for multiple winter temperatures. Consequently, cumulative functions can be fitted to the binomial termination data over time, from which the underlying distributional parameters of diapause termination rates (i.e., mean and variance) can be estimated for each temperature (Fig. 1). We applied this method to the model species *Pieris napi*—a butterfly that diapauses in the pupal stage—revealing a continuous relationship between temperature and diapause progression rate, which decreased with temperature above ~1 °C. Our method further allowed us to partition out the temperature dependence of postdiapause development, which was found to follow the shape of a typical thermal performance curve [TPC, (17)]. In combination, these two temperature-dependent mechanisms form a parsimonious, quantitative framework for explaining the role of temperature in both developmental suppression during fall and winter and in synchronous emergence during spring.

The effect of climate change is often disproportionately strong during winters (18, 19), and winter warming can cause drastic distributional shifts in insects, which can in turn lead to substantial economic and societal damage (20–22). Because our approach can easily be extended to other systems, it can aid in predicting (and thus mitigating) such temperature-dependent distributional shifts (23), marking a significant contribution to the quantitative understanding of insect winter responses.

## Results

### A Right-Skewed TPC Captures Temperature’s Effect on Diapause Termination.

In 2020, we initiated diapause in approximately two thousand Swedish *P. napi* pupae by rearing larvae under short daylength conditions (12:12 h light:dark at 18 °C) (24). After pupation, we split 1,073 diapausing individuals into four constant winter treatments: 2, 4, 8, and 15 °C. Every 4 d, after an initial 60 d of artificial winter, we moved three males and three females from each winter treatment to a postwinter temperature treatment of 20 °C. In 2023, we complemented these data by initiating diapause in approximately 400 additional pupae, split into two new overwintering treatments of −6 °C and 1 °C. From overwintering day 80 and onward, we moved one pupa of each sex from both these winter treatments to 20 °C postwinter conditions each day.

**Fig. 2.**
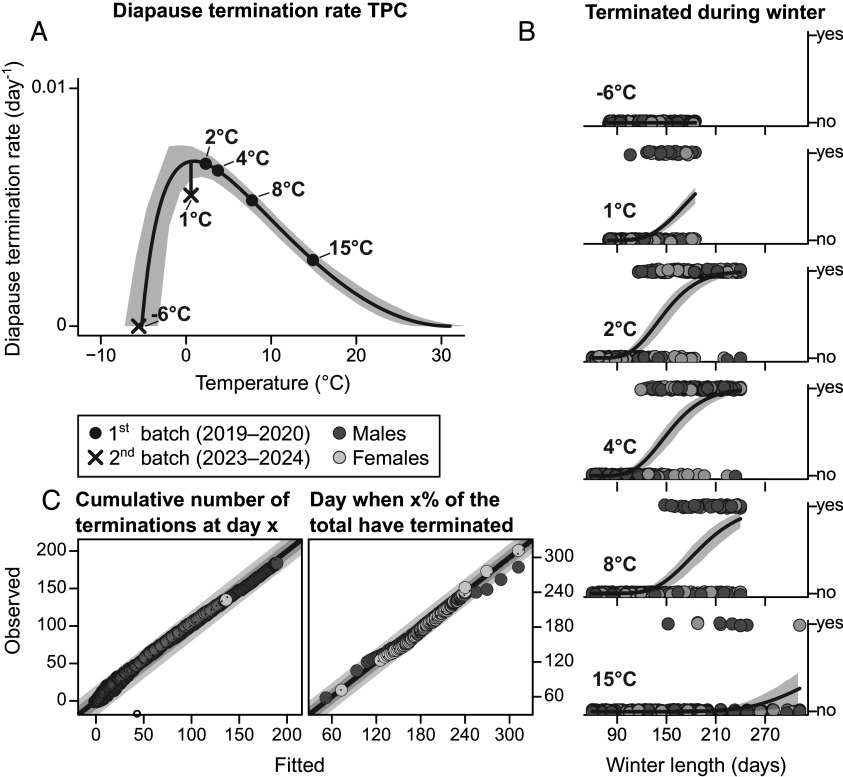
(*A*) The diapause termination rate TPC fitted to binomial data from constant temperature treatments. The curve represents the corresponding TPC of the first experimental batch (used for subsequent predictions), and the vertical line represents the batch effect. (*B*) The resulting inferred cumulative probability functions (lines) for each temperature treatment and the empirical data (circles). (*C*) Assessments of the model fit: x-axes represent fitted values, y-axes represent observed values, full lines represent a 1:1 relationship, and dotted lines show a reduced major axis regression fitted to the data. In the *Left* panel, x and y-values represent a cumulative number of diapause terminations, and each point represents 1 d. In the *Right* panel, x and y-values represent a specific time at which a given proportion of the total terminations have occurred, and each point represents a proportion of the total number of terminations. Dark shaded areas represent an error of 10; light shaded areas represent an error of 20. In (*A*) and (*B*), shaded areas represent 90% HPDIs.

We monitored the pupae in 20 °C daily, recording individuals eclosing within 12 d after the transfer as having terminated diapause. Using a Bayesian framework, we then estimated termination rates in each treatment from the binomial diapause termination as described in Fig. 1. However, based on previous research on thermal effects on *P. napi* diapause termination (25), geometric mean termination rates were not allowed to vary freely across the four temperature treatments, but forced to conform to a right-skewed TPC [(26), Fig. 2 *A* and *B*]. The TPC’s parameters were estimated separately for males and females. Additionally, based on the generally exponential influence of temperature on biochemical rates (27), the variance was assumed to be equal across temperatures on the log scale. Finally, to account for potential differences between the two experimental batches, a batch effect was modeled as an intercept difference on the log scale (*SI Appendix*, Fig. S1).

Comparisons between observations and predictions generated from the fitted model revealed a very good model fit to the empirical data (Fig. 2 *B* and *C* and *SI Appendix*, Figs. S1 and S2), supporting the notion that thermal effects on *P. napi* diapause termination are continuous and quantifiable using a TPC approach. The sex-averaged fitted TPC had a minimum temperature (T_min_) at −5.2 °C (90% highest posterior density interval, HPDI_90_: −7.5 to −2.8 °C), an optimal temperature for maximizing termination rate (T_opt_) at −0.94 °C (HPDI_90_: −0.60 to 2.5 °C), and a maximum temperature (T_max_) at 31 °C (HPDI_90_: 28 to 35 °C). The maximal geometric mean diapause termination rate (R_opt_) differed substantially between the sexes, with males having an R_opt_ estimated to be 0.0075 d^−1^ (HPDI_90_: 0.0067 to 0.0081 d^−1^) and females having an R_opt_ estimated to be 0.0063 d^−1^ (HPDI_90_: 0.0058 to 0.0070 d^−1^), corresponding to diapause termination times at T_opt_ of 134 d and 158 d, respectively. This sex difference indicates that males generally terminate diapause approximately 15% (HPDI_90_: 8 to 24%) faster than females. The sex-averaged SD for the lognormal error distribution was estimated to 0.22 (HPDI_90_: 0.20 to 0.25), corresponding to a percentage difference of 25% (HPDI_90_: 22 to 28%). The estimated TPC with associated uncertainty is visualized in Fig. 2*A*, and sex-specific parameter estimates are presented in Table 1.

**Table 1.** Parameter estimates (posterior modes) with associated uncertainties (90% HPDIs) rounded to three significant figures Asterisks denote parameters that operate on the log scale

			90% HPDI	
Model	Parameter	Estimate (Mode)	Lower	Upper
Diapause termination rate TPC (day^−1^)	T_min_ (males)	−6.18	−8.07	−2.88
	T_opt_ (males)	0.683	−0.892	2.44
	T_max_ (males)	31.3	27.1	35.8
	R_opt_ (males)	0.00746	0.00668	0.00816
	SD* (males)	0.240	0.211	0.281
	T_min_ (females)	−4.11	−8.65	−1.19
	T_opt_ (females)	1.60	−0.851	2.78
	T_max_ (females)	30.9	27.0	35.8
	R_opt_ (females)	0.00634	0.00582	0.00701
	SD* (females)	0.208	0.179	0.240
	Batch effect (intercept)*	−0.230	−0.348	−0.113
Metabolic rate function (Joules × gram^−1^ × day^−1^)	Intercept (males)*	0.273	0.145	0.380
	Intercept (females)*	0.183	0.0856	0.316
	Slope (males)*	0.0926	0.0781	0.105
	Slope (females)*	0.0779	0.0642	0.0911
	SD*	0.177	0.162	0.192
Postdiapause development rate TPC (day^−1^)	T_min_	1.99	1.27	2.77
	T_opt_	29.6	29.1	30.0
	T_max_	36.9	35.5	39.0
	R_opt_	0.152	0.148	0.158
	SD*	0.0852	0.0751	0.0959
	Sex (intercept)*	0.0927	0.0646	0.118

The batch effect describes the difference between the two batches of experimental overwintering treatments (the negative values indicate a lower termination rate in the second batch). The sex effect describes male–female differences (the positive values indicate a higher postdiapause development rate in males).

### The TPC Can Predict Diapause Termination in Fluctuating Temperatures.

**Fig. 3.**
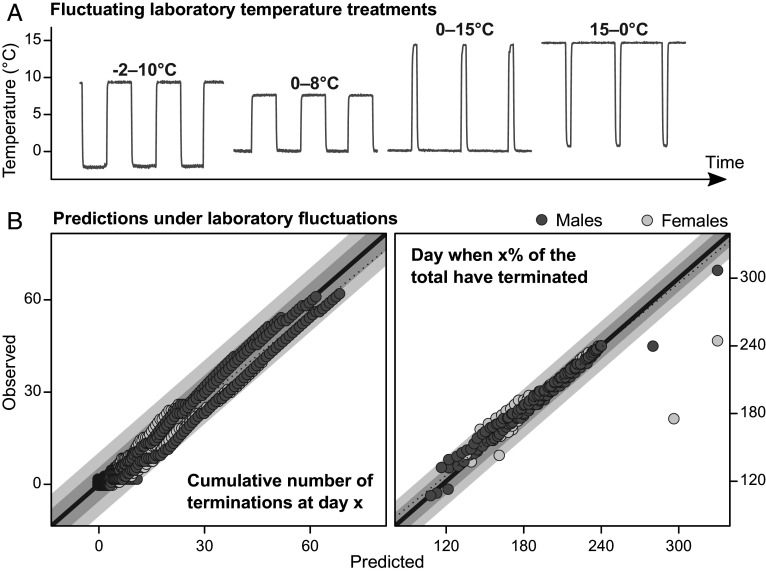
Predictions of diapause termination under fluctuating temperatures from the constant-temperature diapause termination TPC. (*A*) Visualization of the four fluctuating temperature treatments. Lines show temperature data over 3 d (10-min resolution). (*B*) Predictions of diapause terminations in the fluctuating treatments. In the *Left* panel, x and y-values represent a cumulative number of diapause terminations, and each point represents 1 d. In the *Right* panel, x and y-values represent a specific time at which a given proportion of the total terminations have occurred, and each point represents a proportion of the total number of terminations. Prediction accuracies are shown for both sexes in each separate thermal regime. Dark shaded areas represent an error of five terminations or 10 d; light shaded areas represent an error of 10 terminations or 20 d.

To assess the validity of the estimated TPC, we tested its accuracy when predicting diapause termination timing under novel, fluctuating, thermal conditions. In the first experimental batch (2020), we split an additional 1076 of the diapausing *P. napi* pupae into four fluctuating winter treatments (Fig. 3*A*): 0 to 8 °C (12:12 h 0:8 °C), −2 to 10 °C (12:12 h −2:10 °C), 0 to 15 °C (22:2 h 0:15 °C), and 15 to 0 °C (22:2 h 15:0 °C). The pupae were subsequently moved to 20 °C using the same methodology as for the constant temperatures.

By integrating the constant-temperature TPC (with the batch effect set to that of the first batch, Fig. 2*A*) with the fluctuating thermal regimes (Fig. 3*A*), we calculated (sex-specific) expected diapause termination rates for each fluctuating treatment [“rate summation” (14, 28)]. Because the SD is assumed to be fixed on the log scale, the SD estimates from the constant temperature were used together with the expected geometric mean termination rates to predict cumulative diapause termination probabilities (Fig. 1) for each of the fluctuating treatments. When comparing predictions and observations, we found that the constant-temperature TPC yielded accurate predictions of diapause termination timing in the fluctuating treatments (Fig. 3*B* and *SI Appendix*, Fig. S3). The predicted cumulative number of terminations on any given day was within an error margin of 10 terminated individuals (Fig. 3 *B*, *Left*). The day of termination (i.e., the time point at which a given portion of individuals have terminated) was also accurately predicted, with errors generally remaining within a 20-d margin (most being ≤10 d) centered on the 1:1 line (Fig. 3 *B*, *Right*).

### Temperature’s Influence on Diapause Energy Expenditure and Postdiapause Development.

**Fig. 4.**
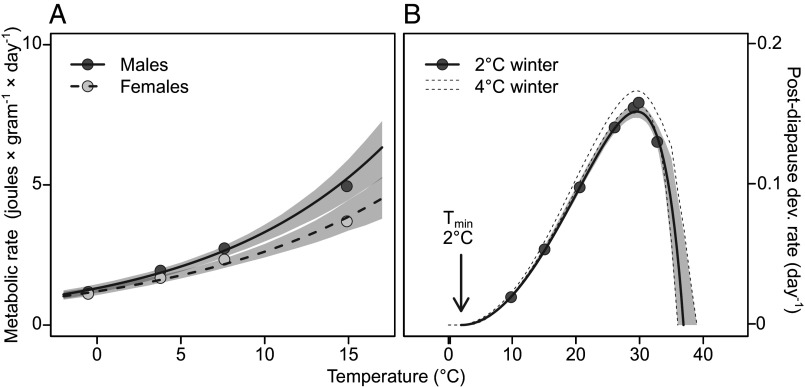
(*A*) The relationship between temperature and mass-corrected metabolic rate. Points represent geometric marginal mean rates. (*B*) The sex-averaged temperature dependence of postdiapause development rates in pupae that had spent approximately 300 d under winter conditions (2/4 °C). Individuals that overwintered in 4 °C displayed slight, but detectable, reductions in postdiapause development times (dashed lines represent the 90% HPDI) when compared to individuals that overwintered in 2 °C. Based on the estimated minimum temperature for postdiapause development of 2.0 °C, the TPC estimated from 2 °C individuals was assumed to represent the true underlying thermal reaction norm for postdiapause termination rates. Points represent geometric marginal means rates of pupae overwintering in 2 °C. Shaded areas represent 90% HPDIs.

To assess the metabolic consequences of overwintering temperature, we performed respirometry measurements (29) of diapausing pupae at the different overwintering conditions, which revealed an exponential effect of temperature on mass-adjusted metabolic rates across the sampled temperature range (Fig. 4*A*). The intercept and slope (for a linear model on the natural log scale) were estimated to 0.27 (HPDI_90_: 0.15 to 0.38) and 0.093 (HPDI_90_: 0.078 to 0.11) for males and 0.18 (HPDI_90_: 0.086 to 0.32) and 0.078 (HPDI_90_: 0.064 to 0.091) for females, respectively (Table 1). Thus, we found an inverse relationship between temperature’s effect on *P. napi* metabolic and diapause termination rates above 1 °C; as temperature rises, diapause termination slows down but metabolic rate increases, revealing a twofold cost of temperature increase on diapause energy expenditure.

After the developmental suppression caused by diapause is terminated, pupae transition to a state of postdiapause development, which progresses when conditions are warm enough (6, 15). To investigate whether the temperature dependence of this postdiapause development resembles that seen in directly developing (nondiapausing) individuals, we split 65 pupae from the 2 °C and 99 pupae from the 4 °C winter treatments across seven different constant temperature postwinter treatments (10, 15, 20, 25, 28, 30, and 33 °C). Postdiapause development rates (day^−1^) were calculated from the time between the transfer to postwinter conditions and eclosion. Importantly, the pupae were transferred after 300 d of winter treatment, ensuring that all individuals had most certainly terminated diapause (Fig. 2 *A* and *B*), thus preventing nonterminated individuals from biasing the estimated postdiapause development rates toward lower values. The distribution of postdiapause development rates across temperatures conformed well to a typical TPC (14) with a lognormal error distribution (Fig. 4*B*).

The T_min_ for postdiapause development was estimated to 2.0 °C (HPDI_90_: 1.3 to 2.8 °C), the T_opt_ to 29.6 °C (HPDI_90_: 29.1 to 30.0 °C), and the T_max_ to 36.9 °C (HPDI_90_: 35.5 to 39.0 °C). The T_min_ estimate of 2.0 °C (Fig. 4*A*) suggests that some postdiapause development occurred in the diapause-terminated individuals kept in 4 °C. Indeed, the estimated average postdiapause development rate was 0.060 (HPDI_90_: 0.031 to 0.085) higher on the natural log scale in pupae overwintering in 4 °C (Fig. 4*A*). This corresponds to a 6% (HPDI_90_: 3 to 9%) completion of postdiapause development under 4 °C winter conditions. Therefore, we assumed that the true R_opt_ (the maximal development rate) corresponded to that seen in pupae kept under 2 °C winter conditions since they could not initiate development before being moved to postwinter conditions. Consequently, the sex-averaged R_opt_ was estimated to 0.15 d^−1^ (HPDI_90_: 0.15 to 0.16 d^−1^), with males developing 10% (HPDI_90_: 7 to 13%) faster than females. This is in accordance with the sex differences in metabolic rates of diapausing individuals, with males expending more energy than females per unit mass (Fig. 4*A*). Parameter estimates are summarized in Table 1.

### Diapause Termination and Postdiapause Development Are Sequential but Thermally Distinct Processes.

**Fig. 5.**
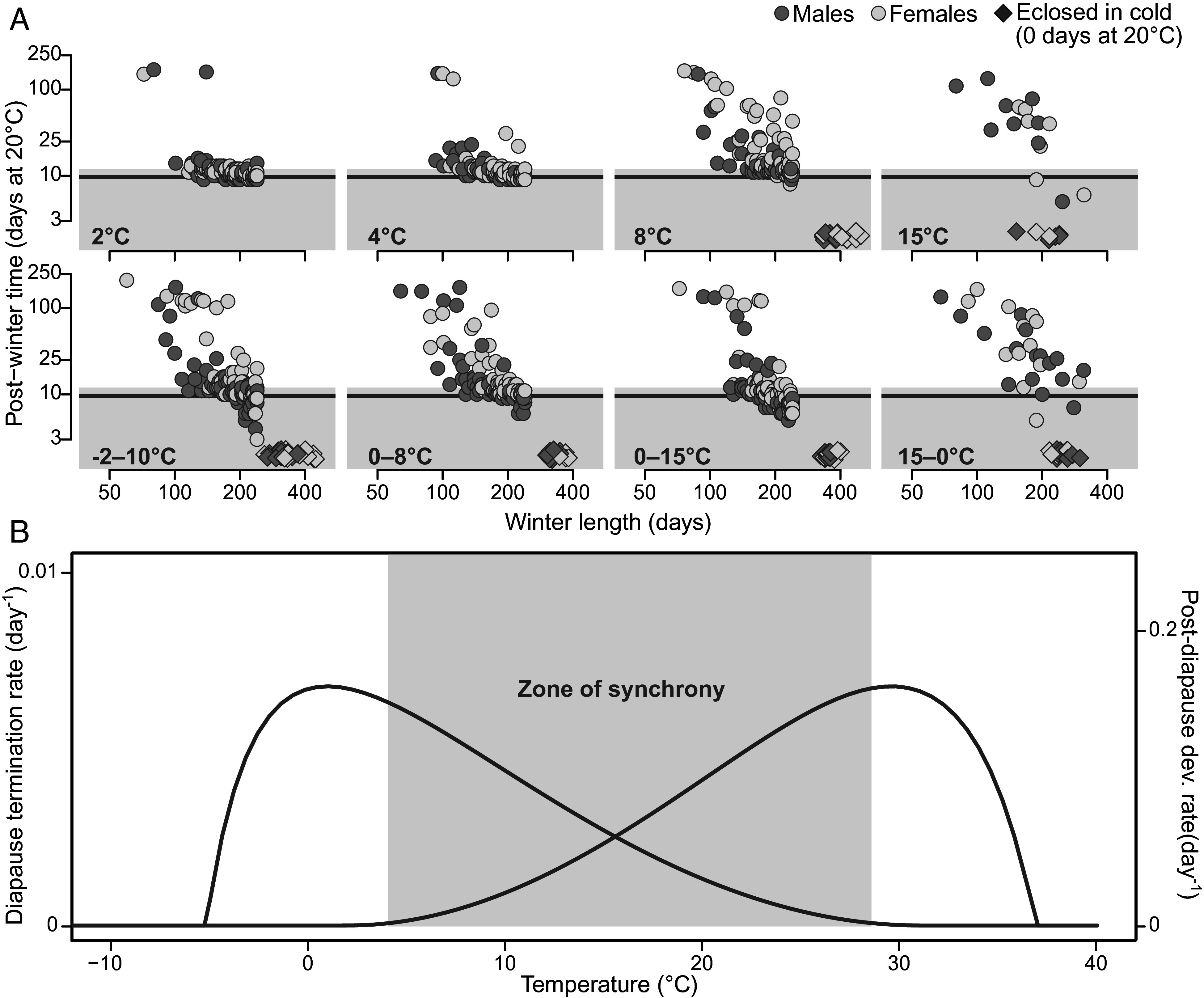
(*A*) The time spent by individual pupae in 20 °C postwinter conditions before eclosing as a function of the time they spent in their respective winter treatment. Dark lines represent the development time corresponding to the geometric mean development rate in 20 °C as estimated by the postdiapause development rate TPC (~10 d). Gray shaded areas represent a postwinter time of 12 d or less, and thus contain individuals that were deemed to have terminated under winter conditions. As winter length increases, the time until eclosion after being moved to 20 °C generally decreases, showing that postdiapause development is sequential to diapause termination. Note that in the 2 °C treatment, where no postdiapause development is expected to occur, postwinter development time is unaffected by winter length. (*B*) Diapause termination and postdiapause as two thermally separated processes with overlap at intermediate temperatures. The left curve and *y*-axis describe diapause termination, and the right curve and *y*-axis describe postdiapause development. The overlap of the two could lead to synchronizing processes, where warm fall temperatures, which would otherwise lead to rapid development, prevent individuals from terminating, and cool spring temperatures prevent individuals from developing, while allowing nonterminated individuals to progress in their diapause termination. This “zone of synchrony” is shown in the gray area.

We found that many pupae which had not fully terminated diapause during winter (i.e., which did not develop into adults within 12 d at 20 °C) could still eclose successfully after prolonged exposure to postwinter conditions (Fig. 5*A*). In other words, diapause termination progressed even at temperatures as high as 20 °C, albeit at a very low rate. Moreover, pupae that had spent a long time in winter conditions sometimes eclosed faster than expected after being moved to postwinter conditions (given the distribution of postdiapause development rates at 20 °C), but this was found only for treatments with winter temperatures permissible for postdiapause development (cf. Figs. 4*B* and 5*A*).

These patterns indicate that some temperatures are permissible for both diapause progression and postdiapause development. This is entirely in line with our expectations given the two estimated TPCs for diapause termination and development rate (Figs. 2*A* and 4*B*), which overlap at intermediate temperatures (Fig. 5*B*). Importantly, the notion of diapause termination and postdiapause development as two sequential and thermally separated processes (Fig. 5*B*) is strongly supported by the fact that some individuals left indefinitely in winter conditions eventually eclosed, but only when those winter conditions permitted fast enough postdiapause development and low enough energy drain during diapause (Fig. 5*A*). Note that relatively warm winter conditions lead to an, on average, slow termination but also to shorter potential postdiapause development times for terminated individuals, which could promote developmental synchrony across different thermal environments [cf. Régnière (30)].

## Discussion

Our results provide clear evidence that diapause termination and postdiapause development in *P. napi* are directly sequential, continuous, and temperature-dependent rate processes with different thermal maxima (Figs. 2, 4*B*, and 5). We quantify winter diapause termination rates across multiple temperatures, showing that the temperature dependence of these rates is well described as a right-skewed TPC with a lognormal error distribution (Fig. 2 and *SI Appendix*, Figs. S1 and S2). We also show that this TPC translates well to fluctuating thermal conditions (Fig. 3 *A* and *B* and *SI Appendix*, Fig. S3).

Our methodology allows for estimating the underlying generative rate process behind binary diapause data while avoiding bias-inducing pitfalls. Previously, the focus of quantitative analysis of diapause duration has been on the portion of individuals that successfully terminated diapause. This is intuitive since only individuals that have completed diapause have a measurable diapause duration. Yet, because interindividual variation in biological rates is usually log-normal, absolute variation in diapause time will be very high at temperatures where diapause progresses slowly (note how the slope becomes flatter in Fig. 1*D*, even as log-normal variance remains constant). As such, diapause termination rates at a given temperature cannot be inferred only from individuals that have successfully terminated diapause since this will introduce a substantial bias toward higher termination rates by filtering out slow-terminating individuals (particularly when most individuals fail to complete diapause). This hurdle is avoided with our methodology.

Predictive accuracy was high for diapause termination in variable laboratory temperatures, but extrapolating to the field might require some additional considerations. First, model uncertainty in warm conditions could hamper predictions when temperature naturally fluctuates around T_max_. Unfortunately, since heat leads to both slow diapause progression and high energetic demands in *P. napi* [Figs. 2*A* and 4*A*; (31)], directly estimating warm-temperature termination is challenging. Additionally, previous works propose a separate diapause initiation phase (6), potentially with a thermal sensitivity different from that of diapause termination (16, 32). An initiation phase with a high temperature optimum might speed up diapause termination in nature, when diapause is initiated during late summer or early fall. However, partitioning the temperature dependence of a potential diapause initiation and subsequent diapause termination is greatly complicated by the cryptic transitions between diapause states. We note that machine learning methods have been effective for visually identifying insect species (33), making use of seemingly cryptic variation (34), and could therefore prove useful for identifying state transitions such as diapause termination.

Our findings contradict the notion of quiescence, defined as exogenously controlled postdiapause developmental arrest (6), as a distinct physiological state. Rather, quiescence appears after diapause in de facto developing individuals just as a by-product of winter’s unfavorable temperatures for development. The sequential nature of diapause termination and postdiapause development—in combination with their separated thermal reaction norms—can make individuals fall from a performance peak (for diapause termination) to a performance low (for postdiapause development) in an instant (Fig. 5*B*). In nature, where microclimatic variation can cause drastic differences in temperature on small spatial scales (35), this could act as a simple mechanism for synchronizing populations. In sites where temperatures are generally low, diapause progresses rapidly but postdiapause development is slow, allowing warmer microclimates to catch up. To date, the ecological impacts of such phenological synchronization mechanisms are poorly known (but see refs. 30 and 36) and constitute interesting topics for future studies.

Moreover, our results highlight the importance of adequate consideration of temporal temperature variation (4, 14). For example, we show that diapause progresses at surprisingly warm temperatures, even as high as 20 °C (Fig. 5*A*). Yet, under constant conditions, such temperatures are unsuitable for diapause termination—diapause terminates extremely slowly (Fig. 2), and metabolic costs are high per unit of time (Fig. 4*A*), leading to resource depletion (24). It is not that diapause cannot progress at these temperatures; it simply cannot finish in time. Still, under naturally variable conditions, diapause progression at warm temperatures likely accounts for a non-negligible portion of the process.

Perhaps, our findings can promote a more nuanced understanding of diapause progression. For instance, unlike previous theoretical models (37), ours does not rely on the concept of declining “diapause intensity” over time. Rather, our approach treats diapause as a constant state of suppressed development which terminates when enough physiological time has passed. The discrete nature of this state transition between diapause and postdiapause does not preclude the underlying process from being continuous, similar to other developmental transitions like molts and metamorphoses (26, 38). We propose that a simple mechanism of two sequential and temperature-dependent rate processes (diapause termination and postdiapause development) with interindividual variation can sufficiently explain empirical patterns of diapause termination in many systems. This eliminates the need for additional variables like diapause intensity or depth while providing a quantitative framework for prediction, compatible with other predictive frameworks [e.g., phenology models (14)]. By describing diapause termination and postdiapause development as temperature-dependent rate processes using TPCs, these processes can simply be integrated with thermal regimes to generate predictions under novel and variable conditions. Moreover, this model provides a parsimonious alternative to old concepts in diapause research. For instance, the term “shallow diapause,” often used to describe an easily terminated diapause (39, 40), can under our model have multiple root causes in nature: The maximal diapause termination rate could be so high that diapause terminates quickly around the optimum, or the reaction norm could be broad so that diapause termination progresses under a wide range of thermal conditions. In one thermal environment, both these reaction norms could yield similar thermal sums, but in another, one reaction norm might lead to more rapid termination than the other. This example highlights that concepts like diapause intensity or depth mean little outside a specific thermal environment. We emphasize that our methodological approach is not limited to cold temperatures and can be applied to investigate these questions in diverse diapause responses, including those with termination occurring rapidly under warm conditions [e.g., Lefevere and De Kort (41), Denlinger (42)].

The view of diapause progression as quantifiably subjected to the passing of physiological time is not new, yet has remained surprisingly uncommon (but see refs. 10, 15, and 16). The model put forth by Johnsen et al. (16) resulted in a reasonable match with emergence dates but did not explicitly estimate any underlying thermal reaction norms. Similarly, the more recent theoretical model by Toxopeus et al. (10) was congruent with their empirical diapause termination data, but the study design used only two temperatures. Thus, while previous works support the notion of an underlying, temperature-driven, diapause termination rate process, they do not formally rule out alternative explanations. Here, we provide robust empirical support for these ideas by explicitly estimating a thermal reaction norm for diapause termination rate using multiple temperatures and then demonstrating its generality across variable environments.

Developing a generalizable quantitative model of diapause progression is significant because it can help understand and predict natural distributions of species limited by their winter biology (9, 23, 43–45). This is particularly important given climate change’s often disproportionate effects on winter temperatures (18, 46, 47), which are believed to facilitate range expansions of in socioeconomically damaging taxa like bark beetles (48). In plants, there are several quantitative models explaining cold-driven vernalization (49), and key genes and pathways involved in vernalization in *Arabidopsis* have been characterized (50, 51). We emphasize that similar studies on the mechanisms behind the responses described here are called for (but see ref. 52 and could provide additional accuracy to predictive models. Moreover, we acknowledge that there is a large diversity of diapause responses, from very brief (53) to multiyear diapause (54), with some species being capable of diapausing in several life stages (55). Therefore, we hope that our model’s generality will be further explored in other important diapause model systems, like *R. pomonella*, disease vectors, like *Aedes albopictus*, and pest insects, like *Ips typographus* or *Dendroctonus ponderosae*. How well our model generalizes across different physiological cold-counting mechanisms remains an interesting and open question.

## Summary

We provide a blueprint for fitting quantitative models of temperature-dependent diapause progression and subsequent postdiapause development. We then apply this method using the butterfly *P. napi*, showing that both processes can be described using TPCs, where diapause termination has a low-temperature optimum and a right-skewed shape, and postdiapause development proceeds in a typical temperature-dependent fashion with a left-skewed response around a high-temperature optimum. We argue that sequential and separated thermal reaction norms like those found in *P. napi* can provide parsimonious explanations for the temperature dependence of diapause progression in many insects. Finally, we note that the generality and physiological basis of our findings are topics ripe for future studies.

## Materials and Methods

### Insect Rearing.

In spring 2019, we sampled 24 gravid *P. napi* females from fields near Stockholm University (59°21′39.4″ N, 18°03′38.0″ E). Out of those females, six laid enough eggs to be used for subsequent crossings. We reared the wild-caught females’ offspring (the F1 generation) in cages with 50 to 100 individuals fed ad libitum with *Armoracia rusticana* under conditions promoting direct development (22:2 h light:dark at 23 °C) (56). We crossed F1 families (reciprocally with regard to sex) to generate six new outbred F2 lines, reared in the same cages and densities as the F1 generation but under diapause-promoting conditions (12:12 h light:dark at 18 °C). We kept the diapausing pupae at 2 °C for 9 mo, which promotes diapause termination but not development (25). Last, we again crossed F2 adults (reciprocally with regard to sex) in 2020 to get six outbred lines for the experimental F3 generation (see *SI Appendix*, Fig. S4 for a pedigree). We reared F3 larvae to pupation in six groups of 400 to 500 individuals in plastic tubs of about 250 dm^3^, with ad libitum access to *A. rusticana*. Four days after we detected the first pupa, we began collecting pupae every fourth day. We kept all pupae under 12:12 h light:dark and 18 °C for 10 d post pupation to standardize diapause initiation conditions. After the initial 10 d, we weighed the pupae and transferred them to different winter temperature treatments.

We sampled another parental generation of insects for respirometry measurements in the same location in spring 2020 and generated an experimental F2 generation via unique crosses of eight F1 families. We reared the F2 individuals to diapause at 18 °C and 12:12 h light:dark and let pupated individuals remain 10 d before weighing and transferring them to temperature treatments in individual Eppendorf tubes with perforated lids. Our rearing was designed specifically to allow for large enough sample sizes while maintaining a balanced genetic structure and avoiding inbreeding—not for directly estimating genetic variation, which was not feasible given the required sample size for the experiment. However, for simplicity, we hereafter refer to the lines as “families.”

Last, to complement the data from 2020, we sampled approximately 20 additional gravid female *P. napi* in late summer 2023 (from the same location as the previous sampling), which were allowed to oviposit freely on *A. rusticana* in a common laboratory environment. The offspring were reared under diapause-initiating conditions (12:12 h light:dark at 18 °C) and moved to winter temperature treatments approximately 2 wk after pupation. Due to resource limitations, no family structure was recorded for these individuals.

### Overwintering Treatments.

For the first experimental batch (2020), we created four constant temperature treatments of 2, 4, 8, and 15 °C to cover the major temperature range that permits diapause termination (based on data from ref. 25). Additionally, we designed four fluctuating treatments to use for validating the model parameterized under constant conditions; a symmetric 0 to 8 °C treatment (12:12 h 0:8 °C), a symmetric −2 to 10 °C treatment (12:12 h −2:10 °C), an asymmetric 0 to 15 °C treatment (22:2 h 0:15 °C), and an asymmetric 15 to 0 °C treatment (22:2 h 15:0 °C). For all treatments, we used Panasonic MIR-154-PE (PHC Europe B.V., Etten-Leur, Netherlands) climate cabinets, except for in the 15 °C treatment, for which we used a Panasonic MLR-352-PE climate cabinet. For the second experimental batch (2023), we created two additional constant temperature treatments of −6 and 1 °C (Panasonic MIR-154-PE cabinets) to better capture the left side of the TPC. We measured temperatures hourly in the cabinets using HOBO MX2202 loggers (Onset Computer Corporation, Bourne, MA). We used actual measured individual mean temperatures for subsequent modeling.

### Diapause Termination Sampling Scheme.

Based on previous research (25), we expected zero terminations during the first 2 mo and therefore began sampling at day 60 in the first batch. We sampled six individuals—three males and three females—every fourth day until day 240, by moving them from winter treatments to a postwinter treatment of 20 °C. In the two slowest treatments (where few pupae terminated diapause), 15 °C and 15 to 0 °C, we extended the sampling period to day 300 by sampling only every 16th day after day 200. In the second batch, sample sizes were lower, and we therefore started sampling at day 80, moving one pupa per day for each sex and winter treatment to 20 °C (12:12 h light:dark), until day 185. Pupae were left in the postwinter treatment until they either eclosed or were deemed to certainly be dead. Those pupae that eclosed within 12 d of being moved to postwinter conditions were recorded as having terminated diapause, and the others as not having terminated diapause.

### Software.

We performed all modeling using Bayesian methods in Stan (57), via the package brms (58) in R [version 4.1.3; R Core Team (59)]. Other important R packages were tidyverse (60), lubridate (61), and bayestestR (62).

### Diapause Energetics.

To estimate the metabolic consequences of overwintering temperature, we adapted a syringe-flow respirometry method from ref. 24. In each constant-temperature overwintering treatment, we measured the metabolic expenditure of a subset of pupae over a 72-h period at day 30, 90, and 150 after pupation. Before each measurement, we weighed the pupae and transferred them individually to sealed 20 mL syringes filled with CO_2_-scrubbed air (scrubbing was done using Ascarite II, Arthur H. Thomas Company, A0403541). We then returned the pupae to their original winter treatments, measuring CO_2_ and O_2_ content in each syringe after 72 h had passed. We measured the gas content using a single line flow setup with CO_2_- and H_2_O-scrubbed air (using Ascarite II; Silica Gel Chameleon® 2-6 mm, VWR chemicals, 83000.290; Drierite, Sigma-Aldrich, 238988-454G). We set the air flow rate to 150 mL min^−1^, with air first passing through a Licor 7000 (Li-Cor, Lincoln, NE) for the CO_2_ baseline and then an FC-2 Differential Oxygen Analyzer (Oxzilla, Sable Systems International, Las Vegas, NV) for the O_2_ baseline. The air then flowed through a syringe containing a pupa, and was again scrubbed of H_2_O using magnesium perchlorate (Sigma Aldrich, BCBK4926V) before passing through the second channel of the Licor 7000 for CO_2_ measurement. Finally, the air was scrubbed of CO_2_ using Ascarite II before entering the second channel in the oxygen analyzer where O_2_ was measured. From these measurements, we converted the units to milliliters per day. We then calculated metabolic rates using the function metabolic rate = vO_2_ * (16 + (5.164 * (vCO_2_/vO_2_))) (63), correcting for individual mass to get the metabolic rate for the insects was calculated and then divided by the individual mass to get Joules day^−1^ gram^−1^.

We modeled the effect of temperature on metabolic rate as an exponential relationship with a lognormal error distribution and sex-specific intercepts and slopes. To account for repeated measures, individual ID was modeled as a group-level effect with random intercepts, and to estimate an average effect across pupal ages, the combined effect of temperature treatment and pupal age was also modeled as a group-level effect with random intercepts for each unique combination of age and treatment. Model fit was assessed graphically and through posterior predictive checks (*SI Appendix*, Fig. S5). For prior specifications and all parameter estimates, see *SI Appendix*, Table S1.

### Postdiapause Development TPC.

To investigate whether normal, temperature-dependent, development resumes directly after diapause, we moved a subset of *P. napi* pupae that had most certainly terminated diapause (300 d in 2 °C and 4 °C, n = 65 and n = 99, respectively) from cold to seven different postwinter temperature treatments (10, 15, 20, 25, 28, 30, and 33 °C). We used Panasonic MLR-352 climate cabinets for the 33, 30, and 15 °C treatments, a Panasonic MIR-154-PE climate cabinet for the 10 °C treatment, and Termaks KBP 6395-L cabinets (Termaks, Bergen, Norway) for the other treatments. Temperatures were logged hourly in each climate cabinet by HOBO temperature loggers, and we used actual measured individual mean temperatures for subsequent model fitting. We recorded individual postdiapause development times in each postwinter treatment, from which individual postdiapause development rates (day^−1^) were calculated. Each treatment included individuals from all lines and both sexes.

We then fitted a TPC to the postdiapause development rate data using The Lobry–Rosso–Flandrois (LRF) function. The LRF function has many desirable properties, like biologically meaningful parameters (T_min_, T_opt,_ T_max_, and R_opt_) and high relative performance compared to other TPC functions (14, 64). We used a log-normal error distribution with a log link function and log transformed the TPC function so that its parameters remained meaningful on the original unit scale. We modeled sex as an intercept effect centered on zero and family as a group-level effect with random intercepts that were allowed to vary with temperature treatment. We modeled historic winter conditions (i.e., 0 or 4 °C) as an intercept effect on the log scale. Model fit was assessed graphically and through posterior predictive checks (*SI Appendix*, Fig. S6). For prior specifications and all parameter estimates, see *SI Appendix*, Table S1.

### Diapause Termination TPC.

Our model of the temperature dependence of diapause termination rate operates under the assumptions that the rates are lognormally distributed, with a unimodal and right-skewed effect of temperature on geometric mean rate. The first assumption is strongly supported by the other empirical distributions of temperature-dependent rate processes in biology, which tend to follow lognormal error distributions [e.g., development rates, metabolic rates, mortality rates; (14, 65, 66)]. The second assumption is based on previous work on *P. napi* diapause termination, which shows that very cold or very warm temperatures are suboptimal for terminating diapause quickly (25).

We modeled the temperature dependence of *P. napi* diapause termination rate using an LRF function [a type of TPC function, (64)], but with a right-skewed shape instead of the typical left-skewed shape. To allow the model to be fitted to binary data, we let the output of the LRF function describe the geometric mean rate of a lognormal distribution, which in turn was used to calculate cumulative distribution over time of the proportion expected to have terminated diapause (Fig. 1). We estimated a common SD for all temperature treatments (i.e., we assumed homoscedasticity on the log scale, which is the norm for temperature-dependent rates). The fit of the LRF function was assessed by comparing estimated cumulative probabilities of diapause with observed binary outcomes assessed under the assumption of Bernoulli distributed errors. Because of potential differences between the experimental batches (e.g., in the first batch, we verified that individuals were alive upon moving them to postwinter conditions—something we, due to resource limitations, could not do in the second batch), we modeled a batch effect as an intercept effect on the log scale. Family variation in diapause termination rate was modeled as a group-level effect with normally distributed (on the log scale) random intercepts that were allowed to vary with temperature treatment (this effect was nullified for the second batch, which had no recorded family structure, through a 1/0 dummy variable). All TPC parameters were estimated separately for both sexes. In summary, our method allowed us to fit a TPC to a binary response variable, measured at different temperatures.

Importantly, to see whether our model assumptions were reasonable, we assessed model fit by comparing observed and fitted cumulative numbers of terminations and running posterior predictive checks (for the latter and prior specifications, see *SI Appendix*, Fig. S1 and Table S1), which revealed a good fit of our model to the data.

### Predictions under Fluctuating Temperatures.

We calculated expected cumulative probabilities of terminating diapause for individuals in the fluctuating temperature treatments using rate summation [i.e., subdividing a variable thermal regime into short segments and summing the segment-specific rates, (67)] based on the estimated diapause termination TPC (for the first experimental batch, Fig. 2*A*) and hourly temperature measurements. We first calculated expected geometric mean diapause termination rates based on individual thermal histories. Then, in combination with the expected variation in diapause termination rates (as estimated by the constant-temperature model), we calculated individual probabilities of having terminated diapause using the cumulative lognormal distribution (Fig. 1). From this, we calculated cumulative distributions of diapause terminations over time at the population level.

Observed and predicted cumulative numbers of terminations were compared to assess predictive accuracy. Observed and predicted cumulative “number of terminations” within a temperature treatment were compared day by day (e.g., how many individuals had terminated after 100 d versus how many individuals were predicted to have terminated at that time). Additionally, observed and predicted “day of termination” within a temperature were compared for each observed increment in proportion of terminated individuals (e.g., how many days it took until 50% of the total number of terminations had occurred versus at what day the model predicted that 50% of the total predicted number of terminations would occur).

## Supplementary Material

Appendix 01 (PDF)

## Data Availability

Experimental data have been deposited in Zenodo (10.5281/zenodo.12634388) (68).
