# Supplementary material for: A quantitative model of temperature-dependent diapause progression
Source: Proc Natl Acad Sci U S A. 2024 Aug 28;121(36):e2407057121. doi: 10.1073/pnas.2407057121 (PMC11388385; doi:10.1073/pnas.2407057121)
Supplement: Supplementary file 1 — Appendix 01 (PDF) [file pnas.2407057121.sapp.pdf]

Supporting information

A quantitative model of temperature-dependent diapause progression

Loke von Schmalensee[1,2]*, Philip Süess[1]*, Kevin T. Roberts[1,3], Karl Gotthard[1,2], Philipp Lehmann[1,2,3]

[1]Department of Zoology, Stockholm University, SE-106 91, Stockholm, Sweden

[2]Bolin Centre for Climate Research, Stockholm University, SE-106 91, Stockholm, Sweden

[3]Department of Animal Physiology, Zoological Institute and Museum, University of Greifswald, D-17489, Greifswald, Germany

***Shared first authorship**

Contents

Figures S1–S6 (pp. 2–7)

Table S1 (p. 8)

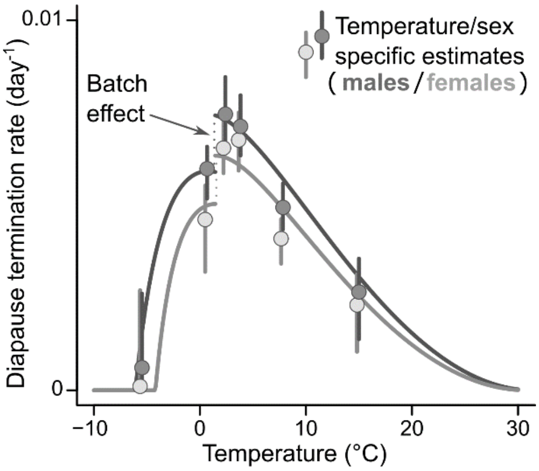

Figure S1. The fit of the diapause termination thermal performance curve model (lines), compared with diapause termination rates estimated using separate cumulative functions for each combination of sex/temperature (circles, bars mark 90% HDPIs). The gap in the curve marked with dotted lines represents the estimated difference between the two experimental batches.

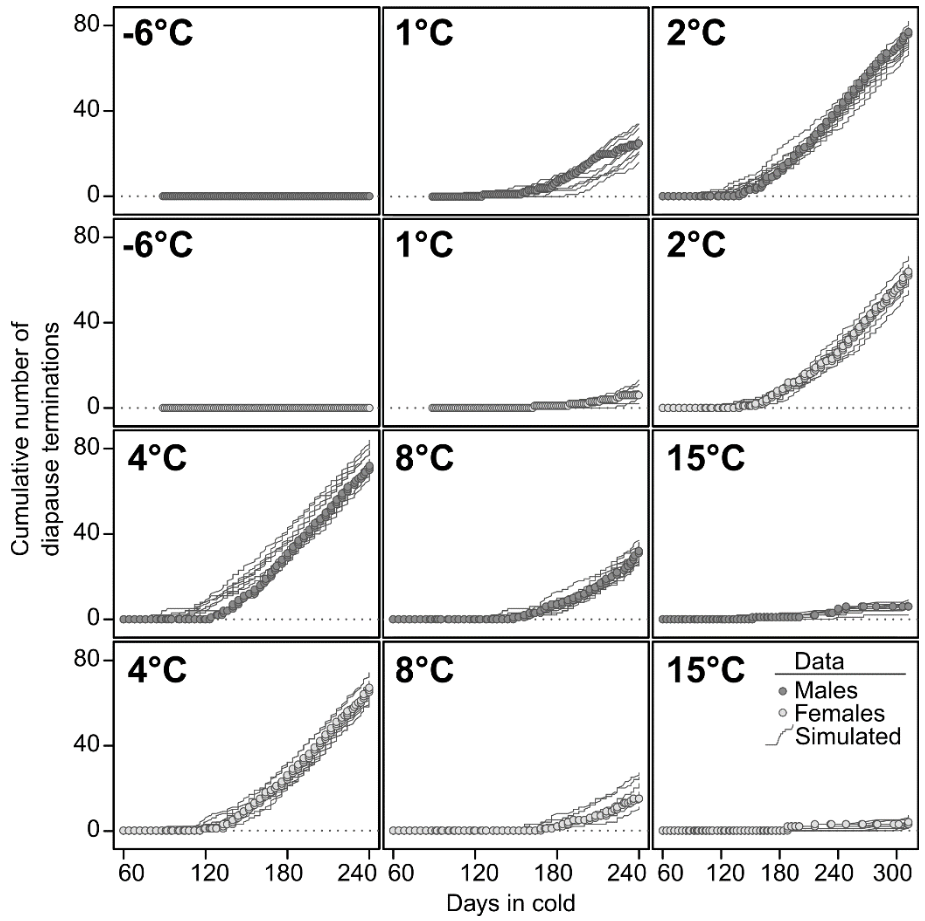

Figure S2. Posterior predictive checks showing a realistic fit of a latent right-skewed thermal performance curve for diapause termination rates to binary diapause termination data. Each grey line represents the cumulative number of diapause terminations over time for each temperature (columns) and sex (rows), calculated from simulated datasets (n = 10) based on random draws from the posterior parameter distributions and residual variation. Points present observed data.

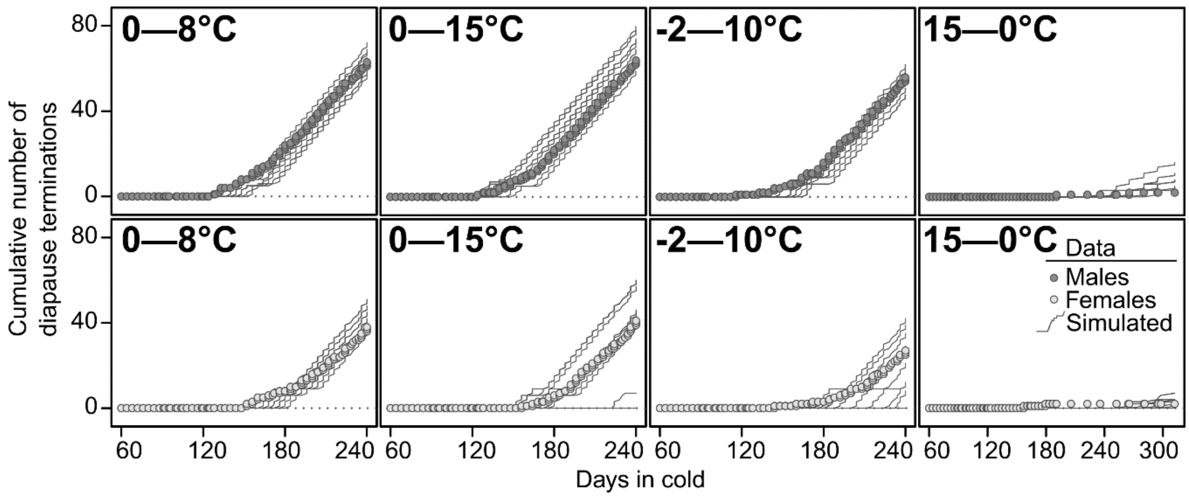

Figure S3. Simulations of diapause termination under variable temperature conditions based on the posterior predictive distribution. Each grey line represents the cumulative number of diapause terminations over time for each temperature (columns) and sex (rows), calculated from simulated datasets (n = 10) based on random draws from the posterior parameter distributions and residual variation. Points present observed data.

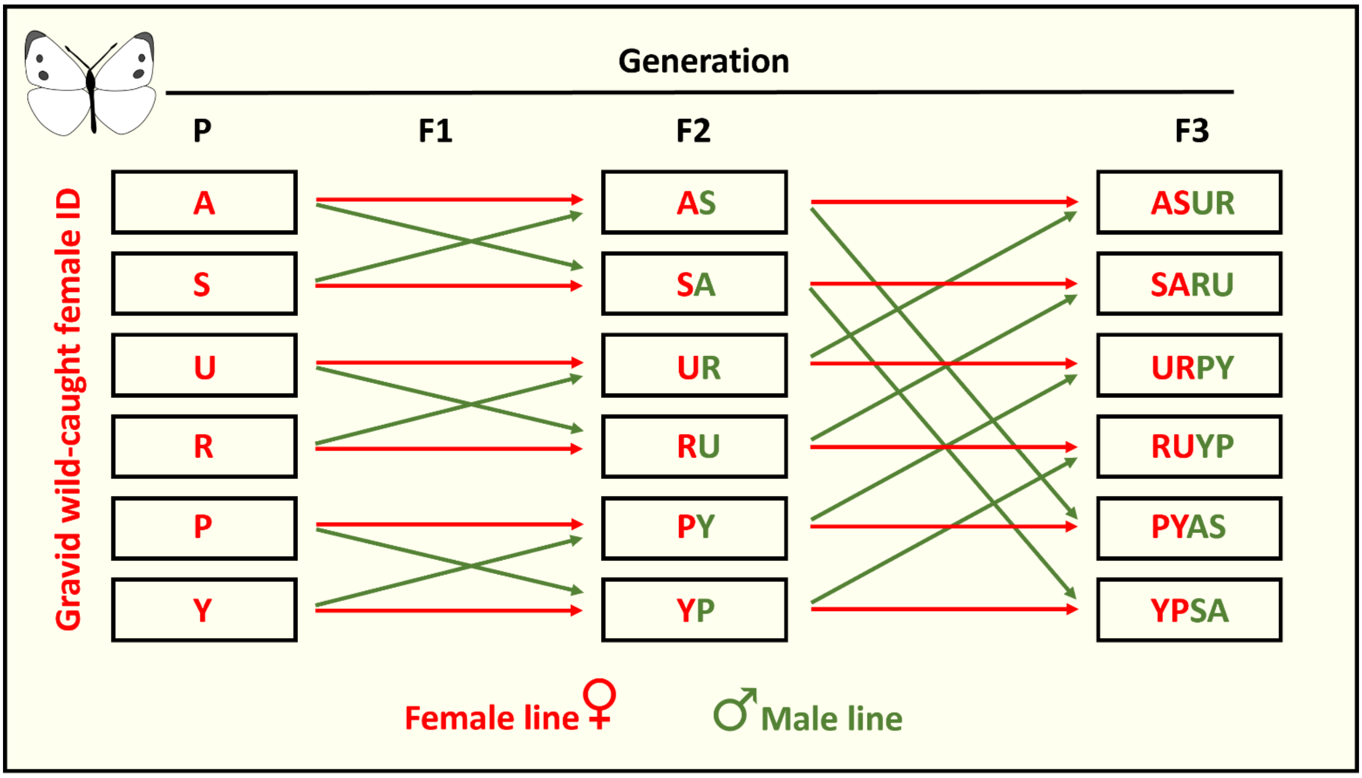

Figure S4. Pedigree chart for the experimental animals.

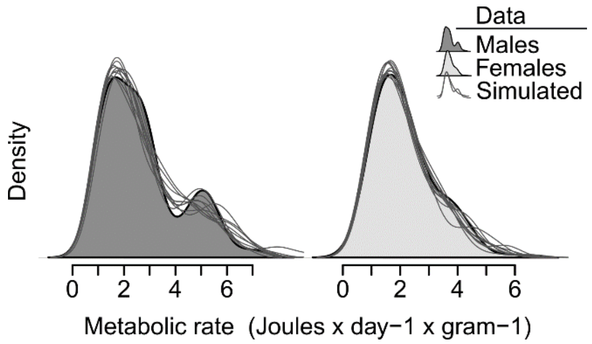

Figure S5. Posterior predictive checks for the metabolic rate model. Shaded areas represent the distribution of observed data, grey lines represent the distribution of data in datasets simulated from the model (n = 10).

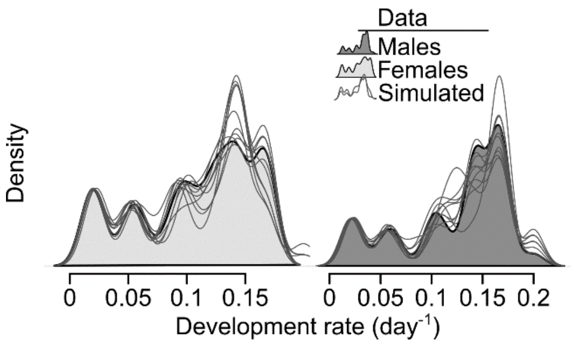

Figure S6. Posterior predictive checks for the post-diapause development rate model. Shaded areas represent the distribution of observed data, grey lines represent the distribution of data in datasets simulated from the model (n = 10).

Table S1. Priors, and parameter estimates (posterior modes) with associated uncertainties (90% HPDIs) rounded to three significant figures. Asterisks denote parameters that operate on the log-scale. The batch effect describes the difference between the two batches of experimental overwintering treatments (the negative values indicate a lower termination rate in the second batch). The sex effect describes male-female differences (the positive values indicate a higher post-diapause development rate in males). The overwintering temperature effect describes the difference in development time in post-winter treatments between individuals that overwintered in 2°C and 4°C (the positive values indicate a shorter post-winter development in individuals that overwintered in 4°C). Daggers denote default priors.

Model	Parameter	Estimate (mode)	90% HDPI lower	90% HDPI upper	Prior
Diapause termination rate TPC (day^{-1})	T_{min} difference (T_{opt}–T_{min}), males	5.87	3.47	8.87	lognormal [mu = log(7.5), sigma = log(1.5)]
	T_{min} difference (T_{opt}–T_{min}), females	5.48	2.83	8.82	lognormal [mu = log(7.5), sigma = log(1.5)]
	T_{opt}, males	0.683	-0.892	2.44	gaussian[mu = 2, sigma = 3]
	T_{opt}, females	1.60	-0.851	2.78	gaussian[mu = 2, sigma = 3]
	T_{max}, males	31.3	27.1	35.8	gaussian[mu = 30, sigma = 4]
	T_{max}, females	30.9	27.0	35.8	gaussian[mu = 30, sigma = 4]
	R_{opt}, males	0.00746	0.00668	0.00816	gaussian[mu = 0.007, sigma = 0.001], zero-bound
	R_{opt}, females	0.00634	0.00582	0.00701	gaussian[mu = 0.007, sigma = 0.001], zero-bound
	batch effect (intercept) *	-0.230	-0.348	-0.113	gaussian[mu = 0, sigma = 1]
	family:temperature st. dev. *	0.176	0.134	0.260	scaled half student-t[d.f. = 3] †
	exp(residual st. dev.) *, males	0.240	0.211	0.281	gaussian[mu = 0, sigma = 3]
	exp(residual st. dev.) *, females	0.208	0.179	0.240	gaussian[mu = 0, sigma = 3]
Metabolic rate function (Joules × gram^{-1} × day^{-1})	intercept (males) *	0.273	0.145	0.380	flat †
	intercept (females) *	0.183	0.0856	0.316	flat †
	slope (males) *	0.0926	0.0781	0.105	flat †
	slope (females) *	0.0779	0.0642	0.0911	flat †
	individual ID st. dev. *	0.0554	0.00823	0.0825	scaled half student-t[d.f. = 3] †
	pupal age : temperature st. dev. *	0.120	0.0860	0.205	scaled half student-t[d.f. = 3] †
	residual st. dev. *	0.177	0.162	0.192	scaled half student-t[d.f. = 3] †
Post-diapause development rate TPC (day^{-1})	T_{min}	1.99	1.27	2.77	gaussian[mu = 0, sigma = 5]
	T_{opt}	29.6	29.1	30.0	gaussian[mu = 30, sigma = 5]
	T_{max}	36.9	35.5	39.0	gaussian[mu = 35, sigma = 5]
	R_{opt}	0.152	0.148	0.158	gaussian[mu = 0.25, sigma = 0.25], zero-bound
	sex (intercept) *	0.0927	0.0646	0.118	gaussian[mu = 0, sigma = 1]
	overwintering temperature (intercept) *	0.0599	0.0314	0.085	gaussian[mu = 0, sigma = 1]
	family:temperature st. dev. *	0.178	0.127	0.250	scaled half student-t[d.f. = 3] †
	st. dev. *	0.0852	0.0751	0.0959	scaled half student-t[d.f. = 3] †
